# Use of VESsel GENeration with Optical Coherence Tomography Angiography and Fluorescein Angiography for Detection and Quantification of Vascular Changes in Mild and Moderate Diabetic Retinopathy

**DOI:** 10.3390/life14070893

**Published:** 2024-07-18

**Authors:** Mariana DuPont, Edmund Arthur, Yazen Shihab, Madelyn Kenny, Swetha Ravichandran, Patricia Parsons-Wingerter, Ruchi Vyas, Matthew C. Murray, Marina Predovic, Shiyin Lim, Nicole Jacobs, Sneha Ramesh, Amanda Vu, Srinivaas Sekaran, Kakarla V. Chalam, Ramana S. Moorthy, Jason Crosson, John Mason, Maria B. Grant

**Affiliations:** 1 Department of Ophthalmology and Visual Sciences, Heersink School of Medicine, University of Alabama at Birmingham, Birmingham, AL 35294, USA; mdupont@uab.edu; 2 Department of Hematology-Oncology, Heersink School of Medicine, University of Alabama at Birmingham, Birmingham, AL 35294, USA; 3 School of Optometry, University of Alabama at Birmingham, Birmingham, AL 35294, USA; earthur@uab.edu (E.A.); mrkenny@uab.edu (M.K.); swetha30@uab.edu (S.R.); 4 Birmingham School of Medicine, University of Alabama, Birmingham, AL 35233, USA; yazen@uab.edu; 5 National Aeronautics and Space Administration (NASA), Washington, DC 20546, USA; patriciaparsonsw@gmail.com; 6 Mori Associates, Ames Research Center, National Aeronautics and Space Administration (NASA), Moffett Field, CA 94035, USA; ruchivyas@gmail.com; 7 Blue Marble Space Institute of Science, Ames Research Center, NASA, Moffett Field, CA 94035, USA; matthewc.murray@hotmail.com (M.C.M.); predovic.marina@gmail.com (M.P.); shiyin.lim.000@gmail.com (S.L.); nicolejacobs@ucla.edu (N.J.); 97.sneha@gmail.com (S.R.); amandavu94@gmail.com (A.V.); sekaransrinivaas@gmail.com (S.S.); 8 Department of Ophthalmology, Loma Linda University Health Care, Loma Linda, CA 92354, USA; kchalam@llu.edu; 9 Associated Vitreoretinal and Uveitis Consultants, Indianapolis, IN 46290, USA; rsmoorthy46032@yahoo.com; 10 Retina Consultants of Alabama Birmingham, Birmingham, AL 35233, USA; jcrosson@uab.edu (J.C.); johnmason@uabmc.edu (J.M.)

**Keywords:** retinal imaging, VESGEN, image processing, fluorescein angiography, optical coherence tomography angiography, vascular segmentation

## Abstract

(1) Background: Previously, VESsel GENeration (VESGEN) software was used to map and quantify vascular changes observed on fluorescein angiography (FA) in subjects (n = 15 eyes) with retinal pathology ranging from mild non-proliferative diabetic retinopathy (NPDR) to proliferative diabetic retinopathy (PDR). In the current study, we used VESGEN for the assessment of individuals with early-stage NPDR imaged by FA (Cohort 1) and by optical coherence tomography angiography (OCTA; Cohort 2). (2) Methods: Cohort 1 included type 2 diabetics (T2D), represented 21 eyes (ranging from no DR to moderate DR), and also included nondiabetic controls (NDC; n = 15 eyes). Cohort 2 consisted of 23 eyes from T2D subjects (including no DR subjects and moderate DR subjects) and NDC (n = 18 eyes). (3) Results: In the FA-VESGEN study, total tortuosity (Tv) of microvessels (G ≥ 6) increased in T2D with mild DR compared to the controls. In contrast, the VESGEN analysis of OCTA images showed that vessel length (characterized as density) was lower in T2D subjects before the diagnosis of DR and following the diagnosis of DR when compared to the controls. Additionally, T2D showed a significant decrease in vessel area (density). (4) Conclusions: FA elucidated the vessel morphology of small-generation microvessels to a greater degree than OCTA; however, OCTA identified changes in vessel density better than FA. VESGEN analysis can be used with both standard FA and OCTA to facilitate our understanding of early events in DR, including before the clinical diagnosis of DR.

## 1. Introduction

Diabetic retinopathy (DR) represents the most common complication associated with diabetes mellitus (DM) and is recognized as the primary cause of blindness in diabetic adults. Thus, early detection, intervention, and treatment are crucial. By 2050, DR is projected to affect 16 million individuals, with 3.4 million experiencing vision-threatening problems or blindness [1,2].

Retinal changes are predictors of systemic vascular pathology, including hypertension [3], diabetes [2,4], renal disease [5], and sickle cell disease [6]. Yet it remains challenging to evaluate subtle changes in retinal blood vessels due to the complexity of the branching arterial and venous trees in the retina [7,8,9,10]; thus, current DR clinical examinations focus on secondary vascular changes such as microaneurysms and hemorrhages [11,12,13]. 

In a previous study conducted by Parsons-Wingerter et al. [14], vascular changes in patients with mild non-proliferative diabetic retinopathy (NPDR) to proliferative diabetic retinopathy (PDR) were quantified. Vessel Generation Analysis (VESGEN) [15], an automated microvascular mapping and quantification software, was used to map and quantify vascular changes in the previous study and indicated that vascular changes occurred in an oscillatory fashion, with vessel length and vessel number increasing in the moderate and PDR stages while being less in the mild and severe DR stages. However, this study had limitations, as it did not include diabetic individuals without clinical evidence of DR or age-matched nondiabetic controls. Also, the FA images were taken with a 55° lens camera which did not provide detail to the macular region, which is better visualized by a 35°lens camera [14]. 

The current study sought to overcome the constraints of the prior study by comparing the DR subjects to diabetics without any evidence of clinical DR and to age-matched nondiabetic controls. We also asked whether examining optical coherence tomography angiography (OCTA) images could provide advantages to FA.

## 2. Materials and Methods

### 2.1. Study Participants

Informed consent was obtained from all subjects involved in the studies. Following informed consent, subjects (control, DM alone and DM with confirmed DR) [16] were recruited from Indiana University (IRB: 1402550709), the University of Alabama at Birmingham (IRB-300009129), and the University of Florida (IRB#535-2011). Grading of NPDR was carried out by 2 masked board-certified retina specialists using standard early treatment diabetic retinopathy study (ETDRS) guidelines [17]. Individuals with mild NPDR had ETDRS levels between 20 and 35, whereas those with moderate NPDR were classified as ETDRS levels 43–47. No subjects had diabetic macular edema. Normal subjects had an ETDRS grading of 10. Exclusion criteria for controls and diabetics were ages less than 18 years, significant lens changes or media opacities that prevented imaging, chronic, or acute infections (HIV, hepatitis, or tuberculosis), any known history of other retinal diseases or diabetic complications other than DR, pregnancy, presence of malignancy, and prisoners. Inclusion criteria for diabetic subjects include diabetes defined as hemoglobin (Hb)A1c ≥ 6.5% with or without DR. Cohort 1: FA images from 21 diabetic eyes no DR (n = 5), mild DR (n = 8), and moderate DR (n = 8) and NDC (n = 15 eyes) (Table 1). Cohort 2: OCTA images from 23 diabetic eyes with no DR (n = 17) and moderate DR (n = 6), which were compared to the controls (18 eyes). One diabetic subject (1 eye) was removed from the study due to a failed screening, resulting in a total of thirteen diabetics (23 eyes). Baseline demographics of the entire cohort were comparable in all groups and are shown in Table 2.

### 2.2. FA Image Acquisition

Retinal imaging was conducted on all subjects using a 35° lens field. FA was performed using an intravenous injection of a 5 mL 20% sodium fluorescein. Retinal imaging was then conducted on all subjects with a SPECTRALIS imaging system using a 35° lens field. The photographers were intentionally blinded to the severity of the retinal condition of each participant, ensuring an unbiased data collection. High-quality, mid-phase images were obtained after complete arteriovenous filling of the posterior pole. Images with the maximum capillary plexus detail devoid of/with limited dye leakage were selected for each eye for further analysis.

### 2.3. OCTA Image Acquisition

Macular-centered OCTA images, that were 20 × 20°, of the superficial vascular plexus (SVP) consisting of 512 B-scans, 512 A-scans per B-scan, 12-micron spacing between the B-scans, and 5 frames averaged per each B-scan location (Spectralis HRA + OCT; Eye Explorer version 1.10.4.0; Heidelberg Engineering Heidelberg, Germany) were obtained for 30 participants (13 DR patients and 17 controls) for a total of 41 eyes. The superficial vascular plexus (SVP) was defined as the composite retinal vasculature from the inner limiting membrane (ILM) to the inner plexiform layer (IPL)/inner nuclear layer (INL) boundary [18,19]. The signal quality values of all the OCTA images from the vendor software ranged from 30 to 40, with 40 being the highest possible value that can be obtained.

### 2.4. Image Processing

Each grayscale FA and OCTA image was processed manually in Photoshop (Adobe, Inc., San Jose, CA, USA) to a binary (black/white) image together with a region of interest (ROI) image, subject to inspection by at least one expert reviewer. Vascular branching in the binary images was separated into arterial and venous vascular patterns (FA only) using color fundus images for FA images based on previously established characteristics [7]. Each binary image of arterial or venous pattern, together with its ROI, served as the input images for the VESGEN analysis described below. Due to poor image quality, one control (FA) and two diabetic (one FA and one OCTA) retinal images were removed from the study. FA images of the controls were previously used for a prior study on retinal vessel changes in pulmonary arterial hypertension [15]. Total vessels were analyzed for OCTA imaging due to the inability of distinguishing arteries from veins. 

### 2.5. Vascular Quantification

VESGEN analysis (Bethesda, MD, USA), a JAVA-based software for interactive use, was employed to analyze the vascular features of the FA and OCTA images [14,15,20]. This software works as a plug-in to the ImageJ software from the U.S. National Institutes of Health, Bethesda, MD, USA, and is available upon request from the U.S. National Aeronautics and Space Administration (NASA, https://software.nasa.gov/software/ARC-17621-1 (accessed on 15 December 2023; recently reviewed). VESGEN has several analysis options [21]. 

The results in VESGEN 2D are provided in pixel units. The software uses a mix of standard ImageJ processing algorithms and custom algorithms unique to the software. For example, it offers automatic methods for detecting regions of interest, including a method to identify the midpoint boundary between a selected vessel region and other non-selected vessels. VESGEN 2D utilizes specialized algorithms to determine branch boundaries and assign branches to their respective branching generations (G1, …, Gx) for the analysis of vascular trees.

Users can interact with VESGEN 2D to optimize the output analysis. After analyzing and mapping a vascular tree into distinct generations, the vessels can be combined into groups such as small, medium, and large branching generations (see below). This helps to facilitate further analysis. The output results include vascular maps illustrating the generational assignments for vascular trees and distance maps displaying local vessel diameter, as well as the ROI used for calculating the vessel density parameters.

VESGEN was employed in this study to examine all the FA and OCTA images. The analysis considered the following vascular parameters: vessel tortuosity (Tv), vessel length density, and vessel area density. The VESGEN software allows for the automated classification of blood vessels into generation(G)s 1–3 (large) and G4–5 (medium) according to user decisions. Vessels of generation 6 and smaller are considered microvascular vessels (G ≥ 6). The VESGEN vascular tree option was used to analyze images displaying branching, asymmetric, nonhomogeneous structures with tapering vessels, such as those seen in 35° FA. Additionally, the study utilized the VESGEN vascular tree network composite option for the OCTA images displaying a closed vascular network geometry.

Vessel area density (Av) and vessel length density (Lv) are key metrics for quantifying vascular structures within a region of interest (ROI). Vessel area density (Av) is defined as the density of the total vascular area within the ROI, calculated by dividing the vascular area by the ROI. Vessel length density (Lv) measures the density of the total vessel length. Additionally, vessel tortuosity is assessed using the distance method, which involves dividing the length of the vessel by the distance between its two endpoints. The ROI plays a crucial role in these measurements, as it defines the specific area within which the density functions, such as vessel area density (Av) and vessel length density (Lv), are computed.

### 2.6. Statistical Analyses

Following normalizing of data, the Shapiro–Wilk test of normality was performed to assess the Gaussian distribution of the data, where *p* < 0.05 indicates non-normal/nonparametric data. Grouped data determined to be normally distributed were assessed for statistical significance by an ordinary one-way parametric ANOVA with Holm–Šídák’s multiple comparisons test. Data determined to be non-normally distributed were assessed for statistical significance by a nonparametric Kruskal–Wallis test with a Dunn multiple comparison test. Tukey’s multiple comparison tests with a single pooled variance were also used. An ordinary two-way ANOVA was used when applicable in addition to Tukey’s multiple comparison test with a single pooled variance. Due to the small sample size, the outliers were not removed. Statistical analysis was performed using GraphPad software. A *p* value < 0.05 assessed statistical significance.

## 3. Results

### 3.1. Clinical Characteristics of FA Subjects

**Figure 1:**
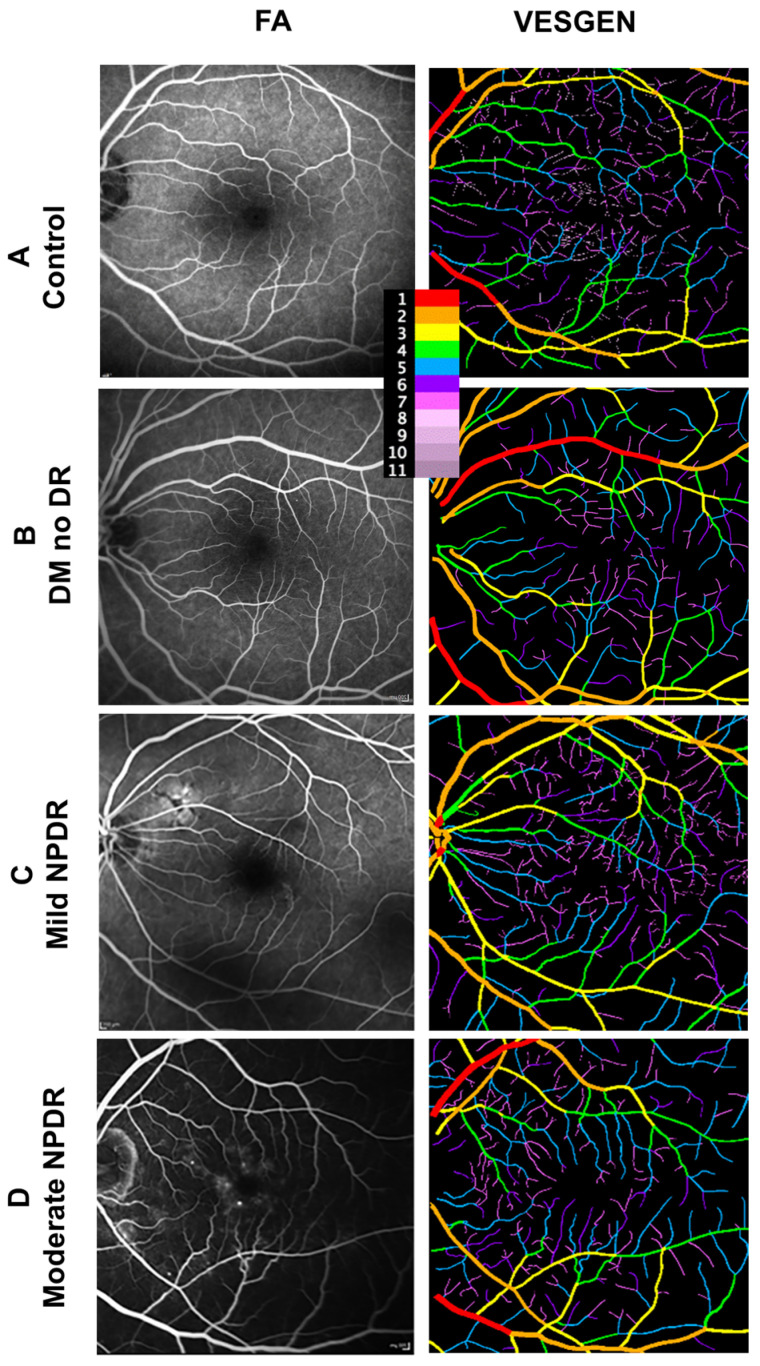
VESGEN characterization of FA images from controls and diabetic individuals. Vascular images and VESGEN maps from the right retina of (**A**) control, (**B**) T2D without DR, (**C**) T2D with mild NPDR, and (**D**) T2D with moderate NPDR. Each row represents increasing severity starting with control (**A**), followed by a T2D individuals with no DR (none; **B**), mild NPDR (**C**), and moderate NPDR (**D**). Legend identifies branching generation (center top). FA = fluorescein angiography.

The clinical characteristics of Cohort 1 are outlined in Table 1. FA images (Figure 1) with a 35° field were processed for the VESGEN input. As depicted in Figure 1, the FA images in the first column were processed and prepared for binarization to be input into VESGEN in the second column.

### 3.2. FA Images Demonstrate Changes in Tortuosity

**Figure 2:**
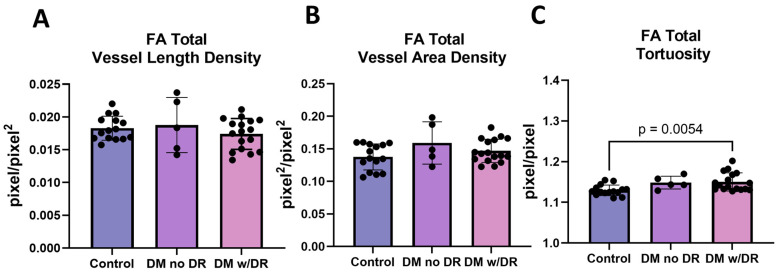
VESGEN analysis of FA diabetics with and without diabetic retinopathy. (**A**) Total vessel length (measured as density) was compared in control, individuals with DM without DR, and individuals with DM with DR. The three cohorts were compared using parametric one-way ANOVA, but no significant differences were found. (**B**) Total vessel area density was compared in the cohorts using one-way nonparametric ANOVA, and no significant differences were found. (**C**) Total tortuosity showed significant differences between controls and DM subjects with DR (*p* = 0.054) when using one-way nonparametric ANOVA. DM = diabetes mellitus; DR = diabetic retinopathy; FA = fluorescein angiography.

Our primary intention of the FA study was to determine if vascular changes were detectable by VESGEN before the onset of clinically diagnosed DR. For this study, individuals were separated based on status as (i) control, (ii) T2D with no history of DR (DM), and (iii) T2D with a history of DR (DM w/DR, Figure 2). When using FA studies, no differences were detected in total vessel length (measured as density) (Figure 2A) and total vessel density (Figure 2B) between the three cohorts. This lack of differences in overall changes in vessel length and densities was confirmed by comparisons of the results for all generations examined, G1–3, G4–5, and G ≥ 6. In contrast, a significant increase in tortuosity (Tv) was detected when comparing the controls to diabetic subjects with DR (*p* = 0.0054) (Figure 2C). FA showed the peripheral retina in more detail and demonstrated increased tortuosity in the T2D subjects with DR compared to the controls.

### 3.3. FA Demonstrates That Arterial but Not Venous Tortuosity Was Increased in Diabetics with DR 

**Figure 3:**
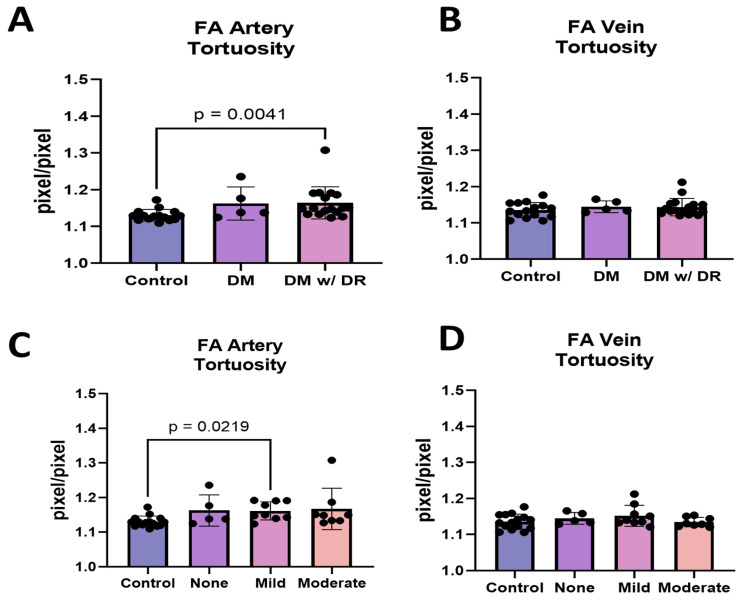
Arterial and venous VESGEN analysis in diabetics with and without DR. (**A**) Arterial tortuosity was compared in the three cohorts. Significant differences were found in the arteries of individuals with DM and DR when compared to controls (*p* = 0.0041). (**B**) Venous tortuosity showed no differences between any of the cohorts. (**C**) Individuals with mild NPDR showed increased arterial tortuosity compared to controls, suggesting that tortuosity may represent a feature of early NPDR (*p* = 0.0219). (**D**) Venous tortuosity did not show a difference between mild NPDR and control. All measurements were performed using a one-way nonparametric ANOVA. DM = diabetes mellitus; DR = diabetic retinopathy; FA = fluorescein angiography.

Because FA detected an increase in total tortuosity, we further explored this by separating the arteries and veins. Diabetics with NPDR had more tortuous arteries but not veins compared to the controls (Figure 3A, *p* = 0.0041). When arteries were further separated into diabetic with mild NPDR and moderate NPDR, arterial tortuosity showed a significant increase between the control and mild NPDR (Figure 3C, *p* = 0.0219), suggesting that tortuosity is an early event in the arteries. No significant differences were observed in venous tortuosity in any of the cohorts (Figure 3B,D).

### 3.4. FA Distinguishes Total Tortuosity by Vessel Generation

**Figure 4:**
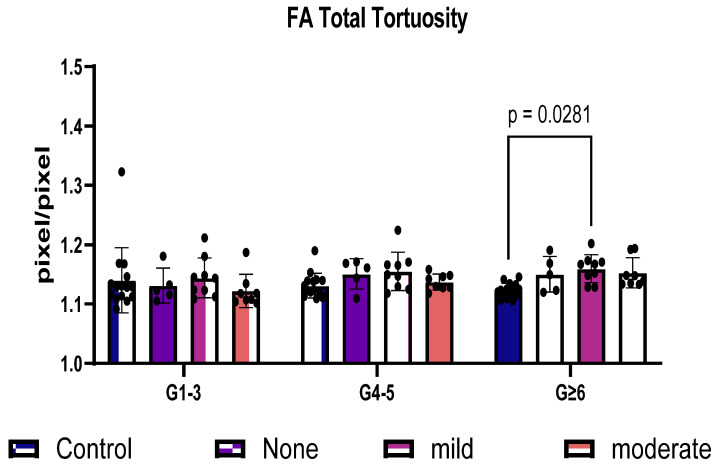
VESGEN analysis between diabetics with and without diabetic retinopathy based on vessel size in FA imaging. Total tortuosity based on vessel generational size was compared between controls and individuals with mild and moderate NPDR. Total tortuosity of FA imaging was analyzed using two-way ANOVA and showed a significant increase in microvessel G ≥ 6 of mild retinopathy (*p* = 0.0281) when compared to controls. DM = diabetes mellitus; DR = diabetic retinopathy; FA = fluorescein angiography.

We next sought to determine whether the size of the vessels impacted tortuosity. Large (G1–3), medium (G4–5), and small (G ≥ 6) total vessels (arteries + veins) were assessed. The tortuosity of the microvessels (G ≥ 6) showed a significant increase (*p* = 0.0281) when comparing the controls and diabetics with mild NPDR but not moderate NPDR (Figure 4).

### 3.5. Clinical Characteristics of OCTA Subjects

**Figure 5:**
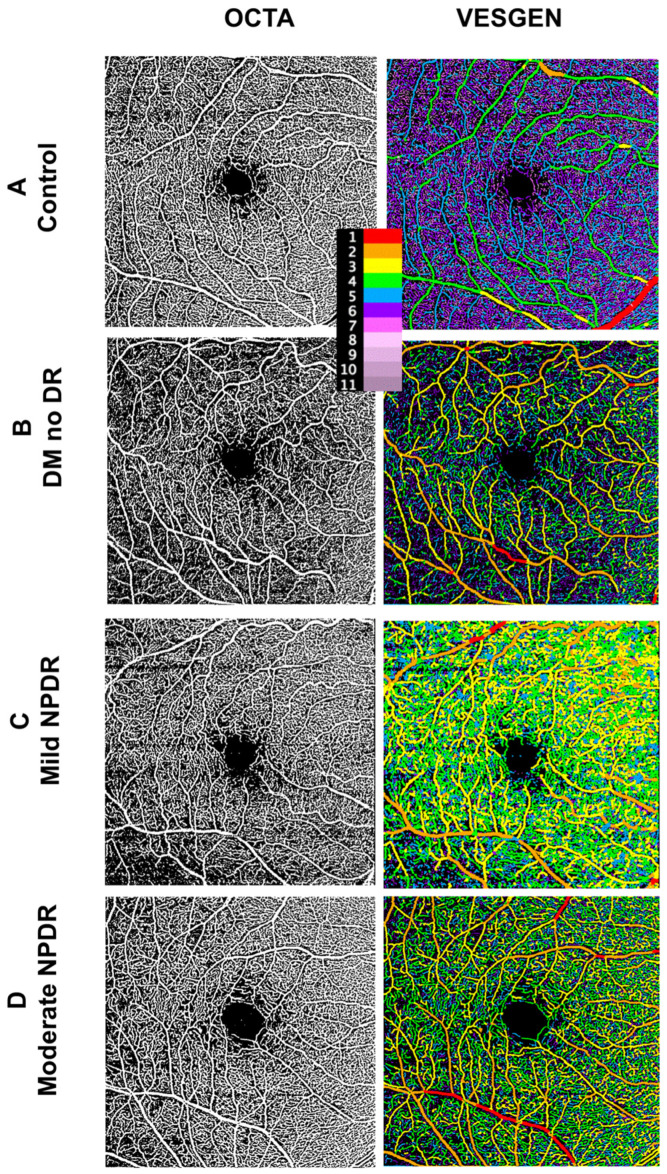
VESGEN characterization of OCTA from controls and diabetes individuals with and without NPDR. OCTA vascular images and VESGEN maps from (**A**) control, (**B**) T2D without DR, (**C**) T2D with mild NPDR, and (**D**) T2D with moderate NPDR (**D**). After the control row, each subsequent row represents increasing severity of DR. Legend identifies branching generation (center top).

The clinical characteristics of the OCTA study subjects are summarized in Table 2. OCTA images (Figure 5; first column) were prepared for input into VESGEN (Figure 5; second column). 

### 3.6. OCTA Images Demonstrate Changes in Multiple Endpoints

**Figure 6:**
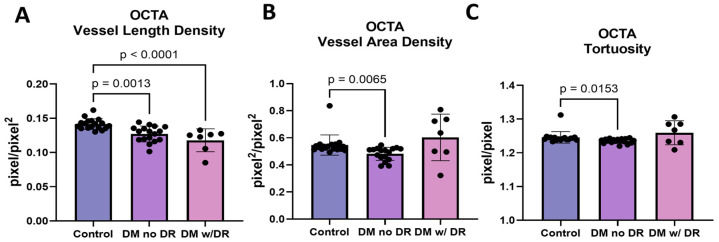
Total VESGEN analysis of OCTA between diabetics with and without diabetic retinopathy (**A**) Total vessel length density was analyzed using parametric one-way ANOVA and showed significant reduction in subjects with DM and no DR and controls (*p* = 0.0013) and a further reduction between DM subjects with DR and controls (*p* < 0.0001). (**B**) Total vessel area density was analyzed using nonparametric one-way ANOVA and showed a reduction in diabetics without DR (*p* = 0.0065). (**C**) Total tortuosity was analyzed using nonparametric one-way ANOVA and demonstrated a reduction in the subjects with diabetes without DR compared to control (*p* = 0.0153). DM = diabetes mellitus; DR = diabetic retinopathy; OCTA = optical coherence tomography angiography.

The use of OCTA indicated a reduction in vessel length density, vessel area density, and tortuosity in diabetics with no retinopathy. Vessel length density showed a significant decrease in diabetics with no retinopathy (*p* = 0.0013) and a further decrease in diabetics with mild or moderate NPDR (*p* < 0.0001; Figure 6A). Vessel area density and tortuosity similarly revealed a significant decrease in diabetics with no retinopathy (*p* = 0.0065; Figure 6B and *p* = 0.0153; Figure 6C, respectively). OCTA focused more on the central retina and indicated a reduction in tortuosity or straightening of vessels before the onset of clinical retinopathy.

### 3.7. OCTA Distinguishes Total Tortuosity by Vessel Generation

**Figure 7:**
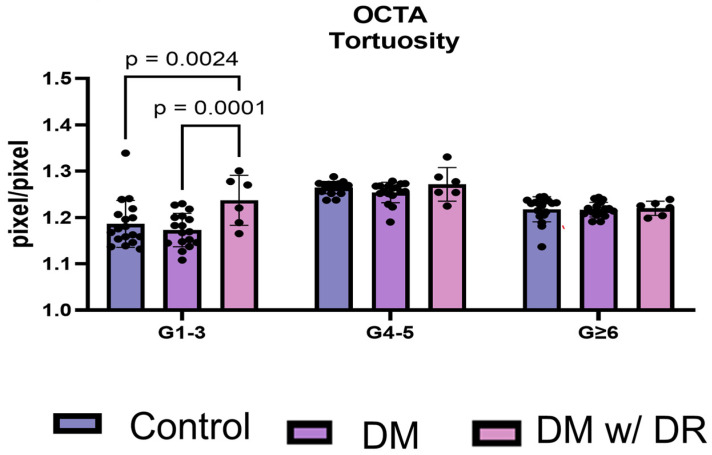
VESGEN analysis between diabetics with and without diabetic retinopathy based on vessel size in OCTA images. Total tortuosity was analyzed using two-way ANOVA and was compared between controls and diabetic individuals with and without DR. Less tortuosity was observed in the G1–3 vessels in controls compared to diabetic individuals with DR (*p* = 0.0024). In T2D individuals without clinical evidence of DR, tortuosity was decreased compared to T2D individuals with DR (*p* = 0.0001). DM = diabetes mellitus; DR = diabetic retinopathy; OCTA = optical coherence tomography angiography.

Lastly, we sought to determine whether the generation of the vessels impacted tortuousity. As previously mentioned, large (G1–3), medium (G4–5), and small (G ≥ 6) total vessels (arteries + veins) were compared. Using OCTA, G1–3 tortuosity was significantly increased in DR compared to the control (*p* = 0.0024) and DM without retinopathy (*p* = 0.0001; Figure 7). When using OCTA, however, the smaller DR microvessels (G4–5 and G ≥ 6) did not appear to change in tortuosity.

## 4. Discussion

In vessel pathology, degeneration and subsequent angiogenesis are hallmark features of DR. Current clinical evaluation of this condition relies on the assessment of microaneurysms and hemorrhages, without the assessment of vascular branching and other morphological changes. Previously, VESGEN was used to measure the “oscillation” of vessel density with the progression of DR in 50° FA [15]. The study found that in DR, there were alternating phases of revascularization and vascular dropout. Initially, changes were observed in the arteries, followed by modifications to the veins. These consistent, progressive arterial changes were found to be more predictive of future DR stages compared to traditional indicators such as microaneurysms, vascular leakage, and cotton wool spots. This suggests that VESGEN analysis could offer improved and more predictive diagnostic capabilities based on early vascular branching changes [15]. However, this initial study did not include a cohort of age-matched controls, nor did it include diabetic subjects without DR. The current study addresses these limitations and uses 35° FA imaging, which facilitates better visualization of smaller microvessels near the macula compared to the 50° FA images used in the initial study. The present study also adds critical information regarding the utility of using VESGEN for the evaluation of OCTA images. 

Both FA and OCTA show that tortuosity changes are observed during the initial stages of DR. However, tortuosity measurements need to compare vessels of the same size rather than the specific generation, as our study supports. FA studies indicate increased tortuosity in the small vessels of generations > six in diabetics with mild DR. OCTA confirmed an increase in tortuosity of the larger microvessels (G1–3); however, these generations correspond to branching levels to the smallest vessels that are detected by FA (G ≥ 6) [22]. Thus, we found that both FA and OCTA imaging showed tortuosity changes, suggesting that the visibility of early-stage DR changes is not preferentially limited to one imaging modality over another. It has been generally appreciated that the tortuosity of retinal vessels is present in advanced diabetes and hypertension [23]; this study is the first to detect differences in early DR. 

Previously, Cui et al. [24] compared OCTA and color fundus photography (CFP) to FA for identifying DR lesions. They found that both methods were equally effective in identifying lesions. Additionally, they suggested that using a combination of OCTA and CFP could provide a less invasive alternative to FA for diagnosing DR [24]. Weinhaus et. al. reported that FA does not image the deeper capillary plexus well; however, this study used nonhuman primate eyes [25]. They suggested that scattering from the deeper retinal layers obliterated the images of the capillaries in the deeper plexus. 

OCTA separates and quantifies the retinal circulation into superficial and deep capillary plexus. The radial capillary network in inner and outer retinal enface zones can be readily imaged by OCTA and decreases in the perfusion of the retinal vasculature easily measured. Sambhav et al. showed that the deeper plexus is involved much earlier than the superficial plexus in DR [26].

Interestingly, in diabetics before the onset of clinically present DR, a reduction was seen in vessel length density, vessel area density, and tortuosity by OCTA. This suggests that OCTA was able to detect changes in vascularity prior to the clinical diagnosis of retinopathy. While examining arteries and veins separately, which can only be achieved using FA, we showed that arteries are tortuous but not veins, which is consistent with previous arteriovenous differences in tortuosity reported by Parsons-Wingerter et al. [14]. 

NASA has developed preliminary automated vessel extraction methods using artificial intelligence (AI), machine learning, and other computational techniques. This process involves extracting binary vascular patterns from grayscale images. The prototype computer code has been incorporated into the VESGEN software, which was launched in 2020. These AI and machine learning models need training and evaluation datasets to learn how to perform specific tasks and have the capability to generalize and perform effectively on previously unseen images. Although AI was not utilized in this study, the aim for clinical application would involve extensive AI training to facilitate the use of VESGEN in clinical practice.

Limitations of our study include that we did not analyze arteries and veins separately in the OCTA images due to our inability to manually separate them, which we were able to do with FA. However, others have used OCT axial reflectance profiles to validate the use of OCTA in guiding artery–vein (AV) classification [27]. Depth-resolved OCT profile analysis enables reliable AV classification, thereby facilitating the early diagnosis of retinal diseases and potentially enhancing deep learning-based AV classification algorithms [27]. Future studies may use these algorithms for the preparation of binary images of arteries and veins from OCTA prior to VESGEN analysis. Another limitation of our study is that the study subjects in the control cohorts were predominantly white, while the T2D subjects included more Black individuals. However, our study was not designed to assess the impact of race on retinal vasculature, and this question will require additional studies with larger cohorts.

## 5. Conclusions

VESGEN analysis software in combination with FA and OCTA imaging can detect differences in tortuosity, vessel area density, and vessel length density. Both 35° FA and OCTA images show similar vascular changes and support our previous findings made with the VESGEN analysis of 50° FA retina scans. Both FA and OCTA combined with VESGEN have the potential to assist in the early detection of subtle vascular changes. VESGEN analysis could distinguish even small improvements with pharmacological or other therapeutic interventions and thus serve as an excellent clinical research tool and can also be used to identify retinal vascular changes in other systemic diseases.

## Figures and Tables

**Table 1:** Baseline characteristics of fluorescein angiography for controls and diabetics.

	FA	
	Control	Type 2 Diabetics
**N**	8	12
**Total eyes**	15	21
Sex *		
Male	4	9
Female	11	5
Age (range) **	25–52	32–69
Race ***		
White	7	5
Black	2	7
Asian	4	0
Other	2	0
**Retinopathy type**		
None	-	5
Mild	-	8
Moderate	-	8
HbA1c% (Mean ± SD) ****	-	8.3 ± 1.8

Age and HbA1c% were determined based on total subjects, while sex, race, and retinopathy were based on total eye count. * 7 patients with unknown sex were not included in the count; ** 4 patients with unknown ages were not included in the count; *** 9 patients with unknown race were not included in the count; **** 6 patients with unknown A1C were not included in count; FA = Fluorescein Angiography, HbA1c% = Hemoglobin A1C, SD = Standard deviation.

**Table 2:** Baseline characteristics of optical coherence tomography angiography for controls and diabetics.

	OCTA	
	Control	Type 2 Diabetics
**N**	17	13
**Total eyes**	18	23
**Sex**		
Male	5	1
Female	13	22
**Age (range)**	46–74	43–65
**Race**		
White	15	2
Black	3	21
Asian	0	0
Other	0	0
**Retinopathy type**		
None	-	17
Mild	-	0
Moderate	-	6
HbA1c% (Mean ± SD) *	-	7.54 ± 1.80 *

Age and HbA1c% were determined based on total subjects, while sex, race, and retinopathy were based on total eye count. * Two individuals had a reading of <8 and were listed as 8 during avg. calculations; OCTA = Optical Coherence Tomography Angiography, HbA1c% = Hemoglobin A1C, SD = Standard deviation.

## Data Availability

The data presented in this study are available on request from the corresponding author.
